# FDG PET/CT Findings of Castleman Disease Assessed by Histologic Subtypes and Compared with Laboratory Findings

**DOI:** 10.3390/diagnostics10120998

**Published:** 2020-11-24

**Authors:** Eun Ji Han, Joo Hyun O, Seung-Eun Jung, Gyeongsin Park, Byung-Ock Choi, Young-Woo Jeon, Gi-June Min, Seok-Goo Cho

**Affiliations:** 1 Division of Nuclear Medicine, Department of Radiology, Yeouido St. Mary’s Hospital, College of Medicine, The Catholic University of Korea, Seoul 06591, Korea; iwao@catholic.ac.kr; 2 Division of Nuclear Medicine, Department of Radiology, Seoul St. Mary’s Hospital, College of Medicine, The Catholic University of Korea, Seoul 06591, Korea; 3 Department of Radiology, Eunpyeong St. Mary’s Hospital, College of Medicine, The Catholic University of Korea, Seoul 06591, Korea; sejung@catholic.ac.kr; 4 Department of Hospital Pathology, Seoul St. Mary’s Hospital, College of Medicine, The Catholic University of Korea, Seoul 06591, Korea; gspark@catholic.ac.kr; 5 Department of Radiation Oncology, Seoul St. Mary’s Hospital, College of Medicine, The Catholic University of Korea, Seoul 06591, Korea; choibo67@catholic.ac.kr; 6 Department of Hematology, Yeouido St. Mary’s Hospital, College of Medicine, The Catholic University of Korea, Seoul 06591, Korea; native47@catholic.ac.kr; 7 Department of Hematology, Seoul St. Mary’s Hospital, College of Medicine, The Catholic University of Korea, Seoul 06591, Korea; beichest@catholic.ac.kr (G.-J.M.); chosg@catholic.ac.kr (S.-G.C.)

**Keywords:** castleman disease, FDG, PET/CT, thrombocytopenia, hypoalbuminemia

## Abstract

Castleman disease (CD) is a relatively rare lymphoproliferative disorder and the pathophysiology of the subtypes are incompletely understood. Fluorine-18 fluorodeoxyglucose (FDG) positron emission tomography (PET)/computed tomography (CT) demonstrates the metabolic activity of inflammatory and tumorous conditions. The FDG uptake intensity and sites of involved lesions on FDG PET/CT were assessed by histologic subtypes, and compared to the patient’s hemoglobin, platelet, albumin, and high-sensitivity C-reactive protein (hs-CRP) levels. In total, 60 PET/CT images of 44 consecutive CD patients were included: 4 (9%) unicentric and 40 (91%) multicentric; 21 (48%) hyaline vascular subtype, 16 (36%) plasma cell, and 7 (16%) mixed or unclassified. The maximum standardized uptake value (SUVmax) and tumor-to-liver (T/L) ratio of involved lymph nodes (LNs) were 5.3 ± 2.4 (range, 1.6–11.5) and 2.8 ± 1.6 (range, 1.1–9.6), respectively, with no significant difference between the histologic subtypes. Higher number of involved LN stations and presence of extra-nodal involvement on FDG PET/CT were associated with thrombocytopenia, hypoalbuminemia, and elevated hs-CRP levels (*p* values < 0.05). FDG-avidity was not different by histologic subtypes and did not correlate with laboratory findings. However, the extent of nodal and extra-nodal involvement as noted on FDG PET/CT was significantly associated with abnormal laboratory findings in patients with CD.

## 1. Introduction

Castleman disease (CD), also known as angio-follicular or giant lymph node (LN) hyperplasia, is a relatively rare lymphoproliferative disorder. The exact incidence of CD is unknown, but previous studies estimated that 6500 to 7700 new cases are diagnosed every year in the US [1,2]. CD has four different histologic subtypes: hyaline vascular, plasma cell, mixed, and plasmablastic. Systemic symptoms and abnormal laboratory findings are reported to be more frequent in the plasma cell subtype. Previous studies demonstrated worse survival outcomes in patients with plasma cell subtype compared to those with hyaline vascular subtype [2,3].

Clinically, CD is classified as unicentric or multicentric. Unicentric CD (UCD) involves a single region of one or more enlarged LNs which can be cured by surgical excision. Multicentric CD (MCD) involves multiple nodal stations and is more frequently associated with systemic symptoms such as night sweats, fatigue, or weight loss. Recently, MCD has been further subdivided according to the presence of human herpes virus 8 (HHV8). Only patients with HHV8-associated MCD have shown the plasmablastic subtype [2,4]. Many patients with HHV8-associated MCD are human immunodeficiency virus (HIV)-positive. Patients without HHV8/HIV infection are also referred to as idiopathic MCD. Excessive proinflammatory hyper-cytokinemia, often including interleukin (IL)-6, is recognized to have a role in the pathogenesis of MCD. HHV8 is the well-established cause of the hyper-cytokinemia in HHV8-associated MCD. However, hyper-cytokinemia in idiopathic MCD is poorly understood, and autoimmune, neoplastic, and viral processes have all been proposed as possible etiologies [5,6,7].

CD can be suspected based on image findings and confirmed following excisional biopsy and histopathological evaluation. The classic computed tomography (CT) appearance of UCD is solitary enlarged LN or localized nodal masses. Hyaline vascular subtype shows homogeneous intense enhancement and plasma cell subtype demonstrates less avid enhancement. However, there is no salient CT finding suggestive of CD, which makes the differentiation from reactive nodal hyperplasia difficult [8,9]. MCD can present as nonspecific finding of multiple, enlarged or non-enlarged (<1 cm) LNs [10,11]. Fluorine-18 fluorodeoxyglucose (FDG) positron emission tomography (PET)/CT, reflecting glucose metabolism, is now a well-established imaging modality for evaluation of various malignancies and lymphoproliferative diseases [12,13,14]. CD is categorized as a benign disorder but known to show increased FDG uptake. Several reports with small sample sizes have reported that FDG PET/CT is more sensitive than enhanced CT and can aid in more accurate staging and response assessment of disease [10,11,15,16,17,18].

The purpose of this study was to evaluate the FDG PET/CT findings of CD by histologic subtypes and compare the FDG PET/CT findings with laboratory findings representative of disease activity.

## 2. Materials and Methods

### 2.1. Patients

All patients with CD (*n* = 30) or benign lymphoproliferative disease (*n* = 256) as diagnosis codes visiting the Department of Hematology at our institution from January 2006 to June 2018 were included. Patients with histologically confirmed CD were selected, and all 44 consecutive patients underwent FDG PET/CT studies. For this study, a pathologist specializing in lymphoproliferative disorders centrally reviewed all the node specimens. We retrospectively reviewed the FDG PET/CT images. Clinicopathologic variables such as age, sex, clinical and histologic subtypes, treatment, and laboratory findings (hemoglobin, platelet, albumin, and high-sensitivity C-reactive protein (hs-CRP) levels) within a month of FDG PET/CT imaging were obtained from medical records. This study was approved by the institutional review board, and the need for patient consent for this retrospective review of imaging studies and clinical data was waived.

### 2.2. FDG PET/CT Acquisition

All patients fasted for at least 6 h, and blood glucose levels were less than 180 mg/dL before the PET/CT study. FDG (222–555 MBq) was injected intravenously, and scanning began approximately 60 min later. No intravenous contrast agent was administered. Studies were acquired on integrated PET/CT scanners, Biograph Duo and Biograph Truepoint (Siemens Medical Solutions, Knoxville, TN, USA) and Discovery 710 (GE Healthcare, Milwaukee, WI, USA). CT began at the vertex or orbitomeatal line and progressed to the upper thighs or toes. PET acquisition followed immediately over the same body region. The acquisition time was 1.5–3 min per bed position. The CT data were used for attenuation correction, and PET images were reconstructed using a standard iterative reconstruction algorithm.

### 2.3. Image Analysis

All FDG PET/CT images were initially reviewed by two nuclear medicine physicians, each with more than 10 years of experience in interpretation of oncologic FDG PET/CT images, who recorded the sites of suspicious CD involvement. The readers were blinded to the laboratory findings. FDG uptake showing patterns typical of reactive nodal hyperplasia was excluded. Discrete nodal FDG uptake that could not be explained by known common physiological findings was included regardless of the size of the LN. Enlarged LN or conglomeration of LNs were included even when the FDG-avidity was low. The readers visually classified the LNs as FDG uptake positive (intensity visually greater than the patient’s liver) or negative (intensity similar to or less than the liver). Two different nuclear medicine physicians each with over 10 years of experience made repeat reads of the FDG PET/CT images. Discrepant reads were settled by consensus of the four readers. The site of the involved LN was designated as for lymphoma staging: cervical (includes supraclavicular), axillary (includes infraclavicular), mediastinal, hilar (includes peri-bronchial), paraaortic (includes upper abdominal and retroperitoneal), iliac (pelvic), mesenteric, and inguinal (includes femoral) stations. Bilateral involvements were counted as involvement of two stations [10,19]. FDG uptake higher than the liver in the spleen or bone marrow (BM), or lesions outside of the lymphatic system that were not typical of known physiological or benign conditions were also noted.

The maximum standardized uptake value (SUVmax) was measured from each FDG PET/CT study using the same workstation (Mirada XD; Mirada Medical, Oxford, UK). SUVmax was defined as the highest FDG uptake value among all of the involved LNs. As different PET/CT systems were utilized in this study, we obtained the tumor-to-liver (T/L) ratio as a normalization method. The T/L ratio was defined as (SUVmax of the target LN)/(mean SUV in 3 cm diameter sphere in right side of the liver).

### 2.4. Statistical Analysis

Categorical variables were expressed in absolute number and percentage of the cases, and continuous variables were expressed as mean ± standard deviation (SD) with range. The Pearson’s chi-square or Fisher’s exact test was used to evaluate difference in proportions and the Mann-Whitney U test was used for comparison of continuous variables. Per institutional normal range, the cut-off values for hemoglobin was 12.5 g/dl for men and 11.5 g/dl for women. The cut-off values for platelet, albumin, and hs-CRP were 150,000/mL, 3.5 g/dl, and 0.47 mg/dl, respectively. All statistical analyses were performed using SPSS version 26.0 (IBM Corp., Armonk, NY, USA). Differences were considered statistically significant when the *p* value was less than 0.05.

## 3. Results

### 3.1. Baseline Patient Characteristics

In total, 60 FDG PET/CT studies of 44 patients (29 men, 15 women; age 51 ± 14 years, range, 26–78) were included. Of the 44 patients, 32 (73%) had single PET/CT study, 9 (20%) had 2 PET/CT studies, and 3 (7%) had 3 or more FDG PET/CT studies. Thirty-nine FDG PET/CT studies were performed in newly diagnosed CD without history of previous medical treatment or radiotherapy. Twenty-one FDG PET/CT studies were carried out for reassessment of known CD. The number of UCD and MCD cases and the histologic subtypes are shown in Table 1. All patients were HIV-negative in this study.

### 3.2. Visual Assessment of FDG PET/CT

Thirty-three (85%) of 39 treatment naïve FDG PET/CT studies had lesions with FDG uptake greater than the liver on visual assessment (Table 2). No statistically significant difference was noted in the FDG-avidity between hyaline vascular and plasma cell subtypes (*p* = 0.603).

Fourteen of the 39 treatment naïve FDG PET/CT images showed diffusely increased FDG activity higher than the liver in the spleen, and 13 images had diffusely increased activity throughout the BM, with 10 showing increased uptake in both spleen and BM. Additional extra-nodal involvement was suspected in 17 MCD patients based on the consensus of readings: involvement in the lungs, 9; bone (with discrete intense FDG uptake without diffusely increased BM uptake), 4; kidney, 3; adrenal gland, 2; Waldeyer’s ring, 2; soft palate, 1; and subcutaneous layer, 1. All three histologic subtypes were responsible for the cases with suspicious extra-nodal lesions (hyaline vascular, *n* = 7; plasma cell, *n* = 7, and mixed or unclassified, *n* = 3).

Per nodal station analysis, 60 FDG PET/CT studies had FDG uptake in total 361 nodal stations (6.0 ± 3.6 stations per study) and the cervical nodal station was the most commonly involved site (Table 3).

### 3.3. Quantitative Assessment of FDG PET/CT

Of the 60 FDG PET/CT studies, nine were not quantitatively assessable due to low FDG avidity (*n* = 1), absence of target lesion following excision biopsy (*n* = 1), or corrupted DICOM files (*n* = 7). The PET parameters of the 51 FDG PET/CT studies are shown in Table 4. In the treatment naïve PET/CT studies, no statistically significant difference was noted in SUV measurement among the histologic subtypes (*p* = 0.442 for SUVmax, *p* = 0.525 for T/L ratio).

### 3.4. FDG PET/CT Findings Compared to Laboratory Findings

**Figure 1:**
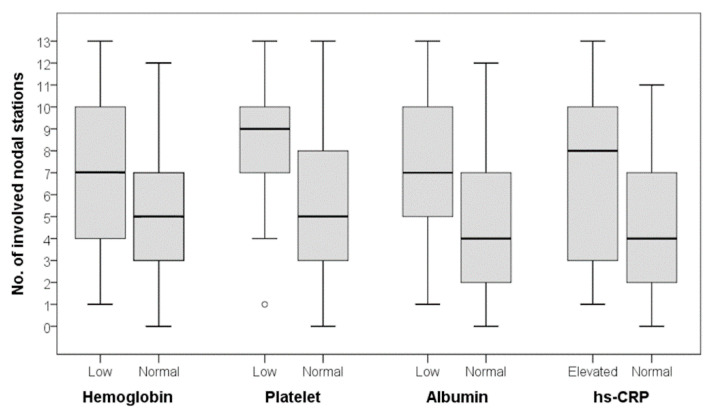
Number of involved nodal stations according to laboratory findings. There was no statistically significant difference for hemoglobin, but significant differences were observed for platelet, albumin, and high-sensitivity C-reactive protein (hs-CRP) levels (Mann-Whitney U test; hemoglobin, *p* = 0.186; platelet, *p* = 0.032; albumin, *p* = 0.027; hs-CRP, *p* = 0.025).

The FDG avidity of the involved CD lesion, expressed as T/L ratio, was not statistically different between the patients with normal and abnormal laboratory findings (Table 5). On the other hand, the number of involved LN stations was significantly higher in patients with low platelet count, low albumin level, or elevated hs-CRP level (Figure 1). Although the patients with low hemoglobin levels showed a higher number of involved LN stations than those with normal hemoglobin levels, the difference did not reach statistical significance.

Of a total of 60 FDG PET/CT studies, 17 showed diffusely increased FDG activity higher than the liver in the spleen, and 20 had diffusely increased activity throughout the bone marrow. Statistically significant differences were noted in the laboratory findings between patients with splenic or BM hyperplasia on PET/CT and those with normal spleen and BM FDG activity (hemoglobin, *p* < 0.001; platelet, *p* = 0.05; albumin, *p* = 0.001; and hs-CRP, *p* < 0.001). Additional extra-nodal involvement was observed in 25 FDG PET/CT studies. Among total 37 cases with suspected extra-nodal involvement on FDG PET/CT, only two cases had normal laboratory profiles. Statistically significant differences were noted in the laboratory findings between patients with any extra-nodal involvement on FDG PET/CT and those without (hemoglobin, *p* = 0.002; platelet, *p* = 0.004; albumin, *p* < 0.001; and hs-CRP, *p* = 0.007).

## 4. Discussion

FDG PET/CT images of patients with histologically confirmed CD were assessed in this study. UCD is reported to be more common than MCD [1,20,21], but the majority of the patients included in our study had MCD (91%). UCD is usually asymptomatic, while patients with MCD often have accompanying systemic symptoms and persistent disease and are more likely to be candidates for imaging. Thus, there was probably a selection bias in our patient population.

Hyaline vascular subtype is reported in 80%–90% of UCD and rarely in MCD, while plasma cell subtype is said to be found in 80%–90% of MCD [22,23]. However, hyaline vascular subtype (*n* = 21) was more common than plasma cell subtype (*n* = 16) in our MCD subpopulation. Other recent studies have shown that the hyaline vascular subtype is not a rare subtype of MCD regardless of HIV infection [24], consistent with our results.

There was no statistically significant difference in the SUV measurement between the two histologic subtypes. A previous study with a small sample size (12 patients), also did not show significant difference in SUVmax between the hyaline vascular and plasma cell subtypes (6.2 ± 5.3 and 4.7 ± 1.8, respectively; *p* = 0.927) [11].

CD can present as palpable mass, weight loss, or feeling of fullness. Patients with MCD can present with flu like symptoms, fever, night sweats, fatigue, or anemia. The treatment of choice is surgical excision for UCD, and most are considered cured. For unresectable UCD, local radiotherapy is an alternative option [25]. Several therapeutic regimens have been reported in MCD with variable efficacy, and there is no gold standard treatment. Wait and watch tactic is chosen in some asymptomatic patients with MCD. Symptomatic MCD is known to have two different clinical courses, steady worsening of symptoms, or bursts of exacerbations that could lead to fatal infections or multiple organ failure [26]. Various empirical systemic treatments based on corticosteroids, immunomodulatory or immunosuppressive agents, cytotoxic chemotherapy (borrowing from regimen for malignant lymphoma), or antiviral agents have been attempted [2,26,27]. Recently, siltuximab (direct monoclonal antibody against IL-6) has become a treatment option for patients with HIV-negative MCD [28].

The aim of therapy would be to minimize the symptoms. Accurate assessment and temporal monitoring of disease activity would guide clinical management. Disease activity has been assessed mostly based on clinical symptoms and laboratory findings (anemia, thrombocytopenia or thrombocytosis, hypoalbuminemia, elevated CRP) [2,29,30,31]. However, there are no established criteria defining response to therapy in MCD, which hampers the interpretation of different treatment modalities [26]. In a previous study of nine patients with HIV-associated MCD, the SUV was significantly higher in patients with active MCD than in patients in remission (*p* = 0.011) [10]. Another study with 24 patients with HHV8-associated MCD showed that nodal SUVmax was statistically correlated with symptom severity, CRP, and HHV8 load [32]. These findings suggested that FDG PET/CT can be an imaging tool to supplement the laboratory findings for evaluation of disease activity and response to treatment in patients with MCD.

**Figure 2:**
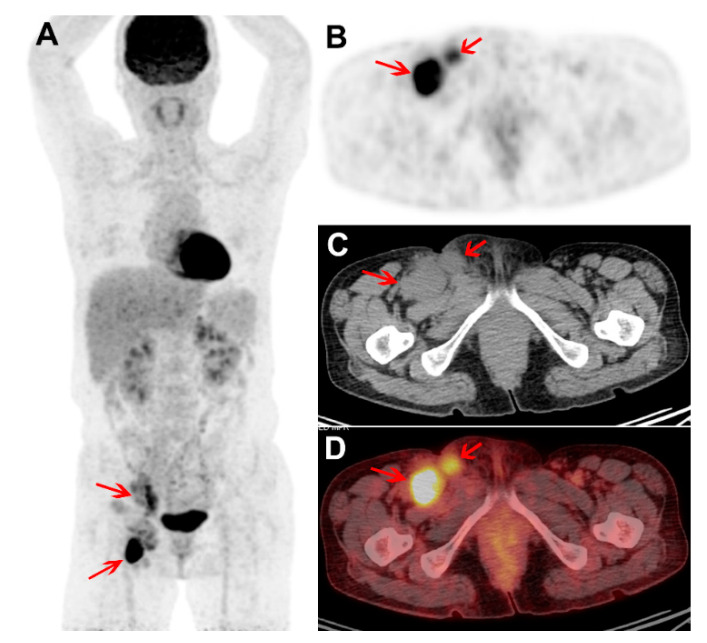
Treatment naïve FDG PET/CT images of a male with plasma cell subtype of multicentric CD (MCD). Maximum intensity projection PET (**A**) and trans-axial PET, CT, and fused PET/CT images (**B**–**D**) show intense FDG uptake in enlarged right external iliac and inguinal nodes (arrows; SUVmax 10.2 and T/L ratio 4.9). The FDG uptake intensity is high, but only two nodal stations were involved. The hemoglobin, platelet, albumin, and hs-CRP levels were within normal range. The patient received chemotherapy and siltuximab. Four years after the PET/CT study, the patient currently remains symptom free.

**Figure 3:**
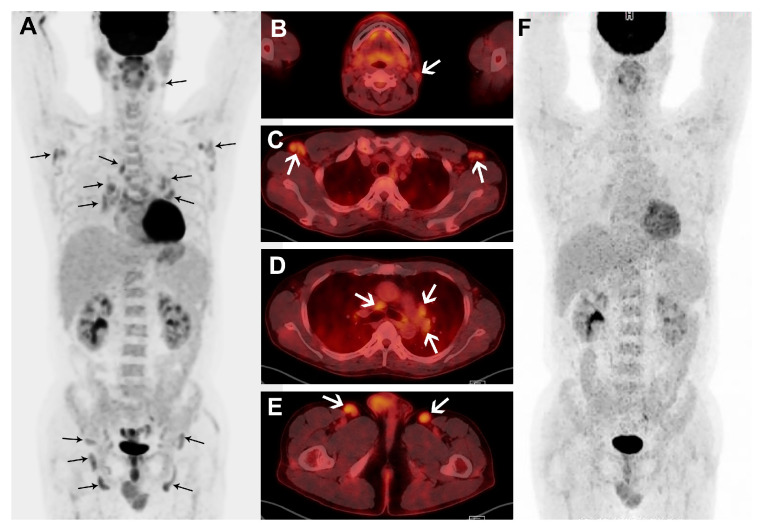
A male patient with plasma cell subtype of MCD confirmed by biopsy of right inguinal node. Maximum intensity projection (**A**) and transaxial fusion images (**B**–**E**) of treatment naïve FDG PET/CT show multiple, mildly enlarged LNs with mildly increased FDG uptake in bilateral cervical, axillary, mediastinal, bilateral hilar, perigastric, bilateral iliac, and inguinal areas (black and white arrows). The patient’s hemoglobin and albumin levels were low, while the hs-CRP level was high. In the FDG PET/CT performed to monitor the response to six cycles of chemotherapy, the nodal FDG uptakes are resolved in the maximum intensity projection image (**F**).

Different PET/CT scanners were included in our study. To overcome this limitation, we calculated the T/L ratio using the liver as internal control to express the FDG uptake intensity. In our results, the T/L ratio showed no correlation with the laboratory findings (Figure 2). However, thrombocytopenia, hypoalbuminemia, and abnormal hs-CRP level were associated with the number of involved LN stations on FDG PET/CT (Figure 3). Patients with low hemoglobin level also had higher number of involved LN stations on FDG PET/CT, though the difference was not statically significant. Among patients with extra-nodal involvement on FDG PET/CT, 92% had abnormal laboratory findings in our study. A recent study with 28 patients with HIV-negative MCD demonstrated that the extent of disease involvement was a significant prognostic factor (5-year overall survival, 91% for disease on a single side versus 73% on both sides of the diaphragm; *p* = 0.03) [24]. These results suggest that determining the full extent of CD may be of value prior to making therapeutic decisions.

Although eventual survival data is lacking, a systematic literature review identified a three-year disease-free survival rate of 45.7% in total of 84 patients with HIV-negative MCD [3]. Recently, four large series of idiopathic MCD and predominantly HIV-negative/HHV8-unknown MCD cases reported five-year overall survival rates of 51%, 55%, 65%, and 77%, respectively [23,24,27,33]. The heterogeneous clinical courses of MCD, limited evidence from clinical trials, and variable treatment regimens probably contributed to the varying survival outcomes. The potential prognostic value of FDG PET/CT in patients with CD are yet unexplored and we are planning further studies looking into the outcome in our patient group.

## 5. Conclusions

FDG PET/CT demonstrated involvement at multiple nodal and extranodal sites in patients with CD. The FDG-avidity was not statistically different among the histologic subtypes. Anemia, thrombocytopenia, hypoalbuminemia, and elevated hs-CRP level were associated with the extent of CD involvement as seen on FDG PET/CT. FDG PET/CT images may be useful for evaluating the disease status in patients with CD.

## Figures and Tables

**Table 1:** Nodal distribution and histologic subtypes of patients with Castleman disease (CD).

		Histologic Subtype			
		Hyaline Vascular	Plasma Cell	Mixed or Unclassified	Total
Distribution	Unicentric	2 (5%)	2 (5%)	0 (0%)	4 (9%)
	Multicentric	19 (43%)	14 (32%)	7 (16%)	40 (91%)
	Total	21 (48%)	16 (36%)	7 (16%)	44 (100%)

No plasmablastic subtype was observed in this study.

**Table 2:** Qualitative analysis of fluorine-18 fluorodeoxyglucose (FDG) positron emission tomography (PET)/computed tomography (CT) in treatment naïve patients with CD.

	Histologic Subtype			
	Hyaline Vascular	Plasma Cell	Mixed or Unclassified	Total
FDG uptake positive	14 (36%)	14 (36%)	5 (13%)	33 (85%)
FDG uptake negative	4 ^a^ (10%)	1 (3%)	1 (3%)	6 (15%)
Total	18 (46%)	15 (39%)	6 (16%)	39 (100%)

^a^ Of the 4 PET/CT studies, one study had no target lesion due to prior surgical excision of the lymph nodes (LN), and the FDG-avidity of the excised LN is unknown.

**Table 3:** Number of FDG PET/CT cases showing involvement by nodal station.

Nodal Station	Treatment Naïve PET/CT	Restaging PET/CT	Total
Cervical ^a^	52 (14%)	24 (7%)	76 (21%)
Axillary ^a^	45 (12%)	21 (6%)	66 (18%)
Mediastinal	20 (6%)	11 (3%)	31 (9%)
Hilar ^a^	27 (7%)	12 (3%)	39 (11%)
Paraaortic	26 (7%)	11 (3%)	37 (10%)
Iliac ^a^	38 (11%)	13 (4%)	51 (14%)
Mesenteric	7 (2%)	3 (1%)	10 (3%)
Inguinal ^a^	35 (10%)	16 (4%)	51 (14%)
Total	250 (69%)	111 (31%)	361 (100%)

^a^ Bilateral involvement counted twice.

**Table 4:** Standardized uptake value (SUV) measurements of the involved LNs.

		Treatment Naïve PET/CT			Restaging PET/CT		
		No.	SUVmax	T/L Ratio	No.	SUVmax	T/L Ratio
			Mean ± SD (range)	Mean ± SD (range)		Mean ± SD (range)	Mean ± SD (range)
Histologic subtype	Hyaline vascular	15	5.1 ± 2.3 (2.7–11.5)	2.9 ± 2.1 (1.2–9.6)	5	7.7 ± 4.8 (4.5–16.2)	4.5 ± 4.1 (1.9–11.6)
	Plasma cell	13	5.5 ± 2.6 (1.6–10.2)	2.8 ± 1.1 (1.5–4.9)	10	5.7 ± 3.6 (1.9–11.3)	2.8 ± 1.9 (1.1–6.6)
	Mixed or unclassified	5	5.6 ± 2.7 (2.5–8.7)	2.8 ± 1.4 (1.1–4.4)	3	4.1 ± 2.1 (2.0–6.2)	2.3 ± 1.1 (1.2–3.4)
	Total	33	5.3 ± 2.4 (1.6–11.5)	2.8 ± 1.6 (1.1–9.6)	18	6.0 ± 3.8 (1.9–16.2)	3.2 ± 2.6 (1.1–11.6)

**Table 5:** Comparison of tumor-to-liver (T/L) ratios and number of involved LN stations with laboratory findings.

		T/L Ratio		No. of Involved LN Stations	
		Mean ± SD	*p* Value	Mean ± SD	*p* Value
Hemoglobin	Low	3.0 ± 2.5	0.203	6.6±3.7	0.186
	Normal	3.0 ± 1.4		5.3±3.4	
Platelet	Low	3.2 ± 1.5	0.343	8.1±3.4	0.032 *
	Normal	2.9 ± 2.1		5.6±3.5	
Albumin	Low	3.4 ± 2.7	0.616	7.1±3.4	0.027 *
	Normal	2.6 ± 1.2		5.0±3.7	
hs-CRP	Elevated	3.2 ± 2.4	0.683	6.8±3.9	0.025 *
	Normal	2.7 ± 1.5		4.3±3.0	

* *p* < 0.05.
